# Abnormal myocardial enzymes in the prediction of mortality and hypertension in COVID-19 patients: a retrospective study

**DOI:** 10.18632/aging.204362

**Published:** 2022-11-02

**Authors:** Shuang Sha, Min Liu, Miaomiao Sun, Long Xiao, Qing Chang, Ying Chen, Jie Huang

**Affiliations:** 1 Shanghai Key Laboratory of Molecular Imaging, Shanghai University of Medicine and Health Sciences, Shanghai 201318, China; 2 Department of Hospital Infection Control, The Fifth Affiliated Hospital, Sun Yat-Sen University, Zhuhai 519000, China; 3 Graduate School of Shanghai University of Traditional Chinese Medicine, Shanghai 200120, China; 4 Yunmeng County People’s Hospital, Yunmeng 432500, China; 5 Clinical Research Center, Jiading District Central Hospital Affiliated Shanghai University of Medicine and Health Sciences, Shanghai 201800, China; 6 Shanghai General Practice Medical Education and Research Center, Shanghai 201800, China; 7 Department of Education and Training Office, Huangshi Central Hospital, Huangshi 435000, China; 8 Department of Critical Care Medicine, Ruijin Hospital Affiliated to Shanghai Jiao Tong University School of Medicine, Shanghai 200025, China

**Keywords:** COVID-19, hypertension, CK-MB, LDH-L, α-HBD

## Abstract

This study aims to determine the differences in myocardial enzymes in COVID-19 patients with and without hypertension. A total of 130 patients with COVID-19 in Yunmeng County People's Hospital were analyzed. The clinical manifestations and laboratory indicators were collected and analyzed. We found that COVID-19 patients with hypertension had higher mortality rate, greater age, and higher rates of basic disease such as diabetes than patients without hypertension. The γ-glutamyl transpeptidase (GGT), blood urea nitrogen (BUN), albumin/globulin (A/G), Ca, Mg, lactate dehydrogenase (LDH), and α-hydroxybutyric-dehydrogenase (α-HBD) levels in COVID-19 patients with hypertension were higher than in COVID-19 patients without hypertension. We found that the predictive effect of the creatine kinase isoenzyme (CK-MB), LDH-L, and α-HBD levels in the COVID-19 patients without hypertension were higher than in COVID-19 patients with hypertension. We used the ROC curve model to predict whether patients would have hypertension, and we found that CK-MB, LDH-L and HBD parameters could distinguish the COVID-19 patients with hypertension and non-hypertension, and could predict the mortality of COVID-19 patients.

## INTRODUCTION

The world population is currently suffering the pandemic disease coronavirus disease 2019 (COVID-19), caused by the novel severe acute respiratory syndrome coronavirus 2 (SARS-CoV-2), which first broke out in Wuhan, Hubei Province, China, in December 2019 [1–3]. COVID-19 spread quickly through other cities in Hubei Province. Many affected patients had a severe acute respiratory syndrome [4] associated with high mortality [5]. The virus was able to create a serious infection, and it was conveyed via person-to-person transmission [6]. As of 2021, the virus has spread rapidly among humans to more than two hundred countries around the world [7, 8]. At present, vaccines are the main means of prevention. Zhou et al. reported that angiotensin-converting enzyme 2 (ACE2) is the receptor by which SARS-CoV-2 enters human cells [9]. ACE2 is highly expressed throughout the body in the respiratory system, blood vessel system, lungs, kidneys, and cardiovascular system [10].

Patients with pre-existing hypertension, diabetes mellitus, and obesity have a considerably increased risk for COVID-19 infection [11]. This association between hypertension and COVID-19 is a global phenomenon. Hypertension was found to be associated with approximately 2.5-fold increased risk of both increased mortality [12]. COVID-19 can also induce myocardial injury and acute coronary syndrome [13]. Understanding the associations between hypertension and COVID-19 infection is important in developing effective targeted therapies [14]. Patients with hypertension are often treated with ACE inhibitors, which causes their levels of ACE2 to increase. Increased ACE2 expression in human cells facilitates COVID-19 infection [15, 16]. Hypertension has been linked to cardiac marker creatine kinase (CK), and high CK is associated with hypertension [17]. Cardiac markers such as creatine kinase (CK), creatine kinase isoenzyme (CK-MB), α-hydroxybutyrate dehydrogenase (α-HBD), and lactate dehydrogenase (LDH) are released into circulation during acute myocardial infarction [18]. Cardiovascular disease, hypertension, CK-MB, and α-hydroxybutyric dehydrogenase (α-HBD) differ significantly between COVID-19 patients who survive and those who do not [19]. Zeng found that in China, hypertensive COVID-19 patients had increased level of LDH, CK and CK-MB [20]. The cardio-metabolic risk factors such as hypertension linked a strong way on the severity of COVID-19. LDH levels and glycaemia mediate the outcome of COVID-19 severity [21]. However, the correlations of these markers with hypertension and lack of hypertension in COVID-19 patients and with mortality are still not fully understood [14].

γ-glutamyl transpeptidase (GGT) and albumin (ALB), ALT, AST are serum biochemical parameters, which can reflect the liver physiological function. A/G, Ca, Mg, P, can reflect electrolyte metabolism in human body. Previous study reported that abnormal liver biochemistry is correlated with increased severity in COVID-19 [22].

In this study, we reported data of COVID-19 patients in the Yunmeng County People’s Hospital in Hubei Province in 2020. The study analyzed 130 COVID-19 patients, and found that the older patients were associated with chronic diseases such as hypertension and diabetes. We found that the biochemical parameters LDH, HBD, GGT, BUN and other microelements had statistical difference between COVID-19 patients with hypertension group and non-hypertension group. Moreover, the CK-MB, LDH, and HBD levels of myocardial enzymes have high predictive abilities in the mortality of COVID-19 patients with non-hypertension. This plays an important role in the diagnosis and treatment of COVID-19 and reduction of mortality. In the non-hypertensive population, once the myocardial enzymes of patients changed, it indicates that the probability of death is increased, and it can be intervened in the early stage.

## RESULTS

### Characteristics of COVID-19 patients with hypertension and non-hypertension

As of May 2020, 130 cases of COVID-19 had been admitted at Yunmeng County People’s Hospital, Hubei Province, including 61 men and 69 women. The average age was 54.36 years old. The patients’ basic information was analyzed (Table 1). The subjects were divided into two groups: those with hypertension and those without. The average age of the COVID-19 patients with hypertension was 60.25±9.11, which is older than those without hypertension: 48.47±15.10 (*P* = 0.0001). The mortality rate of the hypertensive patients (14.29%) was higher than that of non-hypertensive patients (1.96%; *P* = 0.006). Other chronic diseases were also found in the COVID-19 patients. For example, 17.86% of hypertensive COVID-19 patients had diabetes disease compared with 5.55% of COVID-19 patients without hypertension (*P* = 0.01). Results showed that the possibility that a hypertensive patient would also have diabetes is higher than in patients without hypertension. However, the sex ratio, contact history, coronary heart disease, surgical history, respiratory rate, and lung injury status showed no statistically significant difference.

**Table 1:** Basic information of the study population.

**Clinical features**	**Hypertension group** **(n = 28)**	**Non-hypertension group** **(n = 102)**	***P*-value**
Sex (male/female)	10/18	51/51	0.180
Age	60.25±9.11	48.47±15.10	0.0001
Contact history			
Yes	17 (60.71%)	56 (54.90%)	0.583
Uncertainty	11 (39.29%)	46 (45.10%)	
Diabetes	5 (17.86%)	6 (5.88%)	0.01
CAD	2 (7.14%)	4 (3.92%)	0.472
Surgical history	4 (14.29%)	16 (15.69%)	0.856
Pulmonary lesions			
Null	1 (3.57%)	2 (1.96%)	0.223
Unilateral	1 (3.57%)	16 (15.69%)	
Bilateral	26 (92.86%)	84 (82.35%)	
Respiration rate	20 (20, 22)	20 (20,21)	0.713
Mortality	4 (14.29%)	2 (1.96%)	0.006

CAD, coronary artery disease.

The results showed that a few liver and kidney relative factors showed significant difference between COVID-19 patients in hypertension group and non-hypertension group. The serum levels of γ-glutamyl transpeptidase (GGT) (*P* = 0.026), albumin (ALB) (*P* = 0.018), and A/G (albumin/globulin) (*P* = 0.022) levels and blood urea nitrogen (BUN) (*P* = 0.021) were significantly different between hypertension patients and non-hypertension patients (Table 2). However, the serum levels of total bilirubin (TBIL), direct bilirubin (DBIL), indirect bilirubin (IBIL), alanine aminotransferase (ALT), aspartate aminotransferase/alanine aminotransferase (AST/ALT), alkaline phosphatase (ALP), pre-albumin (pre-ALB), and 5-nucleotidase (5-NT) showed no difference. The concentrations of micro elements Ca, Mg, and P were detected. Ca and Mg showed significant differences (*P* = 0.004 and *P* = 0.001, respectively), but that of P did not (*P* = 0.456). LDH (*P* = 0.037) and α-HBD (*P* = 0.041) showed significant difference, and CK and CK-MB showed no significant difference (*P* = 0.389 and *P* = 0.363, respectively).

**Table 2:** Comparative analysis of laboratory examination results.

**Clinical features**	**Hypertension group**	**Non-hypertension group**	***P*-value**
TBIL	9.8 (7.8, 14.1)	10.4 (7.8, 14.8)	0.486
DBIL	3.8 (3.2, 5.1)	3.95 (3, 5.1)	0.962
IBIL	6.9 (4.35, 9.05)	6.35 (4.5, 9.7)	0.736
AST	26 (19.5, 37.5)	25 (19, 34)	0.511
ALT	19 (12.5, 37)	20 (13, 33)	0.600
AST/ALT	1.25 (0.8, 1.9)	1.3 (0.8, 1.6)	0.623
ALP	72 (56.5, 82.5)	70.5 (53, 84)	0.681
GGT	30.5 (22, 45.5)	21.5 (16, 46)	0.026
BUN	4.31 (3.69, 6.05)	3.39 (2.7, 4.45)	0.021
5-NT	9 (7.5, 11)	8 (7, 10)	0.168
TP	69.3 (64.05, 73.7)	70.4 (66.3, 74.4)	0.477
ALB	36.5 (33.25, 40.6)	39.35 (36.6, 41.5)	0.018
GLB	32.35 (29.55,35.55)	32.7 (28.9, 36.0)	0.725
A/G	1.2 (1, 1.425)	1.335 (1.21, 1.68)	0.022
pre-ALB	151 (126.5, 184)	166.5 (129, 210)	0.279
Ca	2.18 (2.11, 2.26)	2.26 (2.19, 2.34)	0.004
Mg	0.74 (0.69, 0.8)	0.8 (0.76, 0.88)	0.001
P	1.035 (0.91, 1.14)	0.97 (0.88, 1.12)	0.456
CK	47 (31, 65)	51 (32, 74)	0.489
CK-MB	24.5 (17, 28)	23 (14, 27)	0.363
LDH	253.5 (209, 306)	203 (174, 267)	0.037
α-HBD	213.5 (194, 267)	184.5 (149.5, 230)	0.041

TBIL, total bilirubin; DBIL, direct bilirubin; IBIL, indirect bilirubin; TP, total protein; ALB, albumin; AST, aspartate aminotransferase; ALT, alanine aminotransferase; ALP, alkaline phosphatase; GGT, γ-glutamyl transpeptidase; BUN, blood urea nitrogen; 5-NT, 5-nucleotidase; TP, total protein; GLB, globulin; A/G, albumin/globulin; CK, creatine kinase; CK-MB, creatine kinase isoenzyme; LDH, lactate dehydrogenase; α-HBD, α-hydroxybutyrate dehydrogenase.

### Predictive mortality of myocardial zymograms in COVID-19 patients

**Figure 1:**
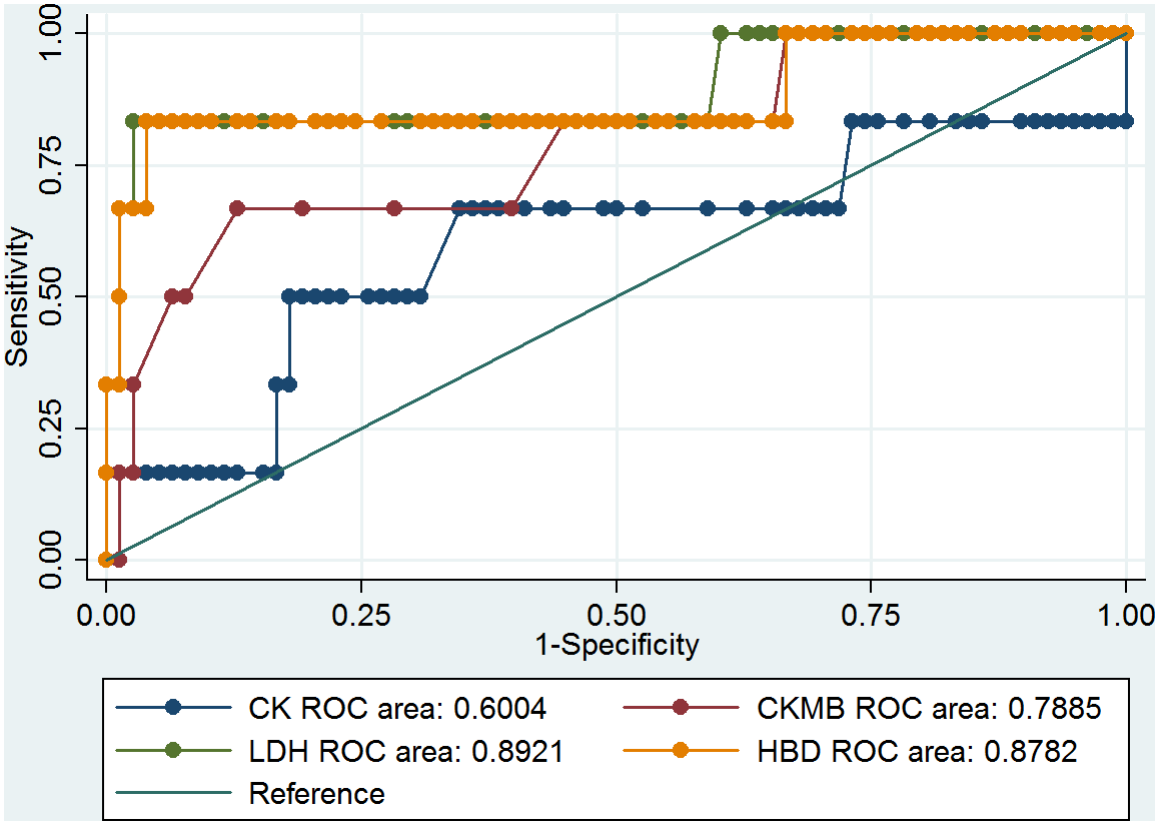
The ROC curves of CK, CK-MB, LDH, and α-HBD and the death of COVID-19 patients.

**Figure 2:**
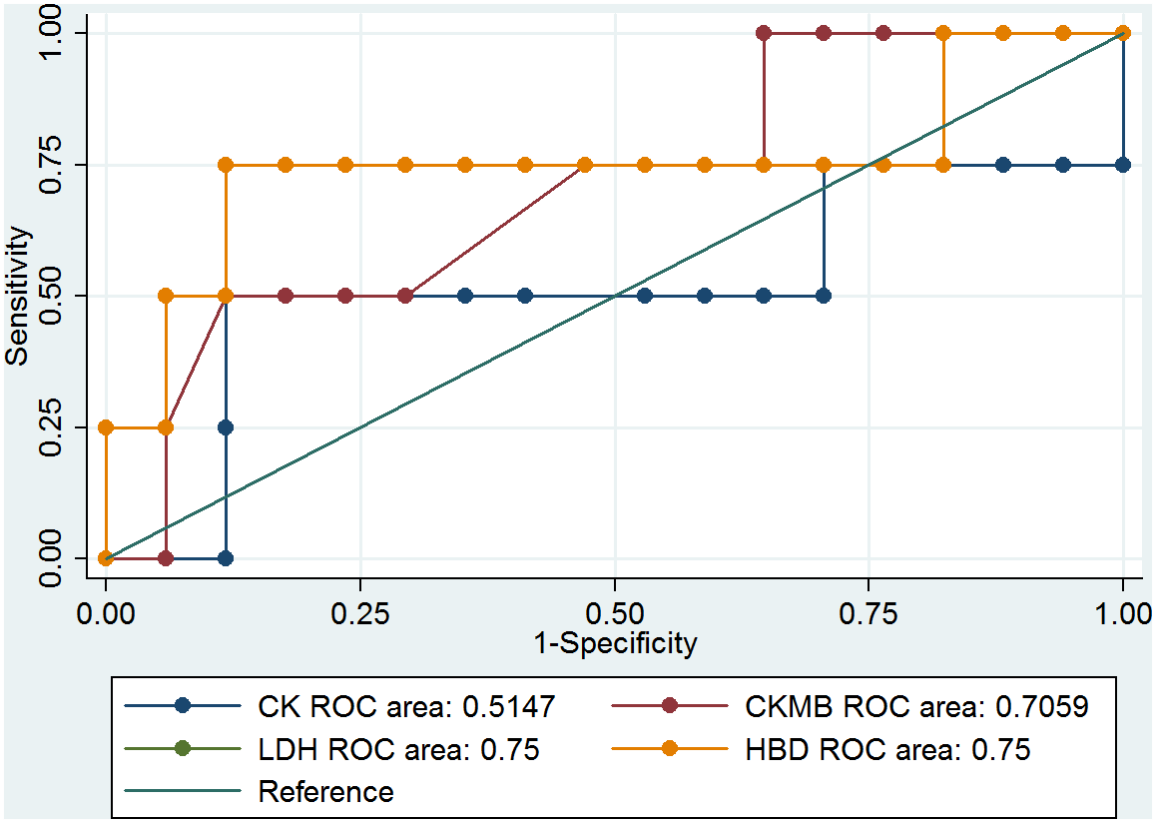
The ROC curves of CK, CK-MB, LDH, and α-HBD in hypertensive COVID-19 patients.

**Figure 3:**
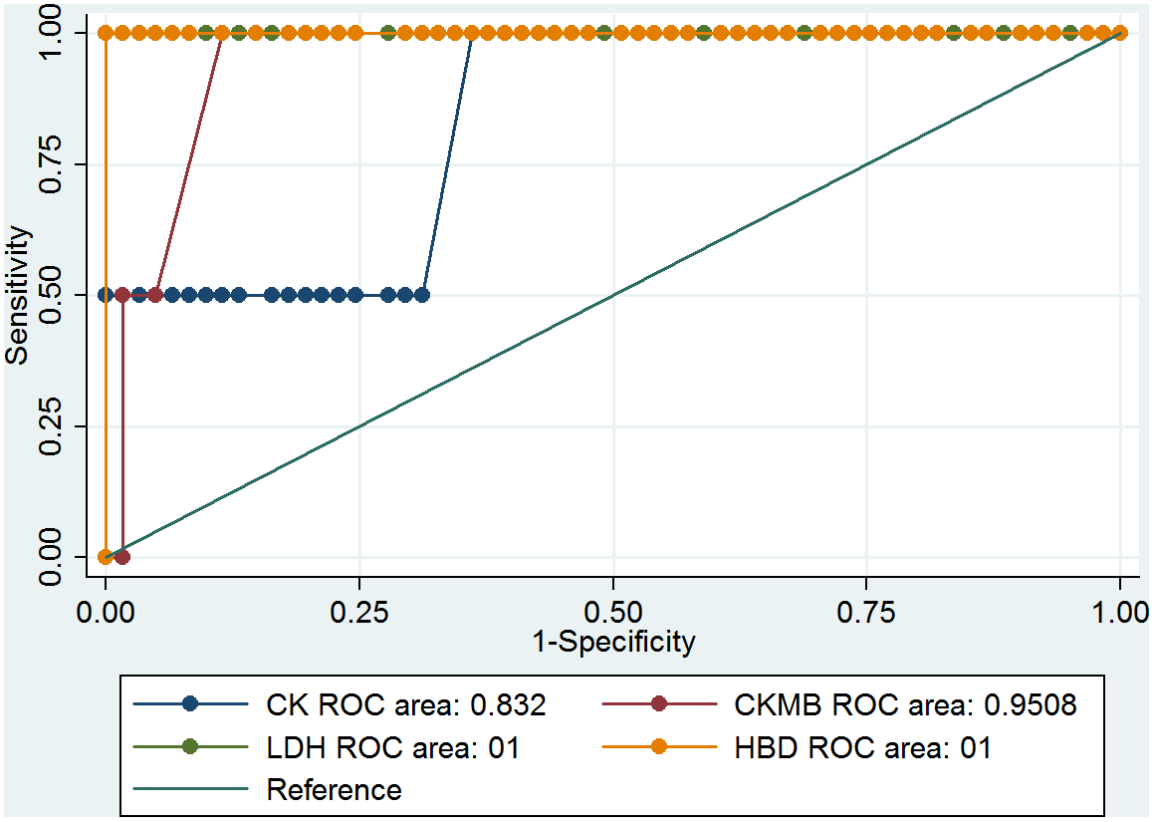
The ROC curves of CK, CK-MB, LDH, and α-HBD in the non-hypertensive COVID-19 patients.

Previous results showed that the cardiovascular system of the COVID-19 patients had been notably damaged. Four parameters, CK, CK-MB, LDH, and α-HBD, of myocardial enzymes could indicate the degree of myocardial damage. The clinical information showed that LDH and α-HBD had significant differences (Table 2), and these results may be correlated with the death rate among COVID-19 patients with hypertension. We analyzed the ROC curve of CK, CK-MB, LDH, and α-HBD, and we found the data can predict the mortality of COVID-19 patients (Figure 1). The ROC curves of CK, CK-MB, LDH, and α-HBD were 0.6004, 0.7885, 0.8921, and 0.8782, respectively (Figure 1). These results showed that LDH and α-HBD can predict mortality of COVID-19 patients better than CK and CK-MB. We also analyzed the prediction of mortality rate in the patients with and without hypertension, respectively. In the hypertensive patients, the ROC curves of CK, CK-MB, LDH, and α-HBD were 0.5147, 0.7059, 0.75, and 0.75, respectively (Figure 2). In the non-hypertensive patients, the ROC curves of CK, CK-MB, LDH, and α-HBD were 0.832, 0.9508, 1, and 1, respectively (Figure 3).

Next, we compared the predictive effect of the area under the ROC curve (AUC) value on the mortality rate in COVID-19 patients with and without hypertension (Table 3). We found that the levels of CK-MB, LDH-L, and α-HBD could predict the mortality rate very well in COVID-19 patients without hypertension. In COVID-19 patients with hypertension, they could predict mortality somewhat well. However, the ability of CK levels to predict mortality in COVID-19 patients with hypertension was low, and its ability to predict mortality in COVID-19 patients without hypertension was moderate. From these results, we concluded that CK-MB, LDH, and α-HBD levels of myocardial enzymes have important predictive abilities in the mortality of COVID-19 patients with non-hypertension.

**Table 3:** AUC value and predictive grade of myocardial enzymes in different groups of patients with COVID-19.

**Variables**	**COVID-19 patient death**			**Hypertension group**			**Non-hypertension group**	
	**AUC**	**grade**		**AUC**	**grade**		**AUC**	**grade**
CK	0.6004	Low		0.5147	Low		0.832	Medium
CK-MB	0.7885	Medium		0.7059	Medium		0.9508	High
LDH-L	0.8921	Medium		0.75	Medium		1.0	High
α-HBD	0.8782	Medium		0.75	Medium		1.0	High

AUC, area under the ROC curve; CK, creatine kinase; CK-MB, creatine kinase isoenzyme; LDH-L, lactate dehydrogenase-L; α-HBD, α-hydroxybutyrate dehydrogenase.

## DISCUSSION

COVID-19 is a pandemic constantly mutated virus spread throughout the world. It adapts to infect humans, and poses global public health threat in the human population [23]. The COVID-19 RBD can bind the cell surface receptor ACE2, which shows high expression in cardiac myocytes and other organs. The ACE2 enzyme plays a key role in the controlling blood pressure of human cardiovascular system [24]. The COVID-19 invades human cardiovascular system and causes myocardial injury. Patients with hypertension or cardiovascular diseases are more likely to increase severe symptoms if infected with COVID-19 [25].

[26, 27] We also analyzed the serum levels of γ-glutamyl transpeptidase (GGT), albumin (ALB), and albumin/globulin (A/G) levels and blood urea nitrogen (BUN) in COVID-19 patients. GGT and BUN levels were higher in the COVID-19 patients with hypertension than in those without hypertension. However, the levels of ALB and A/G were lower. Other studies also reported the serum GGT, AST, and ALT to be significantly higher in patients with severe COVID-19 than in those with mild or otherwise non-severe COVID-19 [28]. The ALB levels of patients with severe COVID-19 were lower than those of patients with mild cases or good outcomes [29]. However, our results showed that the AST, ALT, and AST/ALT levels in the hypertensive and non-hypertensive COVID-19 patients did not differ significantly.

Cardiac biomarkers CK, CK-MB, LDH, and α-HBD were assessed to evaluate heart function. Li et al. found that elevated level of cardiac markers showed increased higher mortality than those normal serum levels [5]. Angelo Zinellu et al. found that higher CK-MB concentrations were associated with the mortality and severe disease in COVID-19 patients [30]. But we found that CK-MB (*P* = 0.363) and CK (*P* = 0.489) had no significant difference in the prediction effect of the mortality. Lizzy M. Brewster et al. considered that CK was linked to hypertension and bleeding risk [17], but our results showed that CK to have a low predictive value of hypertension and mortality cases in patients with COVID-19 and a medium predictive value in COVID-19 patients without hypertension.

Lactate dehydrogenase (LDH) is one of the biomarkers which are determination of prognosis in patients with COVID-19 [31]. In the past, high LDH levels have been associated with worse outcomes in patients with other viral infections [32]. Previous study reported that the LDH levels are significantly different in COVID-19 patients without severe diseases [33]. Other researchers found that the LDH levels were significantly elevated in COVID-19 patients with more severe cases [34]. We had the same results that LDH (P = 0.037) and α-HBD (P = 0.041) values significantly increased in the hypertension patients compared with non-hypertension patients who were infected with COVID-19.

To evaluate the prediction of serum levels of CK, CK-MB, LDH, and α-HBD cardiac biomarkers in the COVID-19 hypertension and non-hypertension patients, ROC curves were analyzed (Figures 1–3) [17]. The prediction value of CK-MB, LDH-L, and α-HBD in the non-hypertensive patients was high, whereas in the prediction of mortality cases and hypertension was medium. In the non-hypertensive patients, CK-MB was found to have a strong predictive effect. It plays an important role in the diagnosis and treatment of new cases of coronary heart disease and in reducing mortality. In non-hypertensive patients, changes in myocardial enzymes indicate a higher probability of death. If clinicians pay close attention, it may be possible to intervene early. The reason the AUC value of CK-MB is lower in the hypertensive group than in the non-hypertensive group may be that the cardiovascular systems of patients with hypertension have been damaged, which may in turn damage the myocardial enzymes; whereas the non-hypertensive group is not affected by this damage, and the changes in myocardial enzymes may be more directly related to death by coronavirus infection (Table 3).

Overall, our analysis of COVID-19 patients with and without hypertension indicated that hypertensive COVID-19 patients were older in age and had a higher mortality rate than their non-hypertensive counterparts. The abnormality of cardiac biomarkers CK-MB, LDH, and α-HBD showed strong predictive mortality abilities in COVID-19 patients without hypertension but not in those with hypertension. The serum levels of GGT, BUN, CK-MB, LDH, and α-HBD were higher in patients with hypertension than in those without. However, our research also had limitations. It is difficult to analyze the value of the blood pressure related to mortality due to the fewer death samples. In the future, we focus on the validation of the correlation of the LDH and α-HBD values with the age of hypertension patients. Larger samples of other hospitals are needed to verify our conclusions. Moreover, the studies were from China and may not be suitable for other populations. Additional investigations are needed to advance targeted treatments and improve patients’ prognosis.

## MATERIALS AND METHODS

### Study population and data collection

All recruited subjects had confirmed cases of COVID-19. A total of 130 patients positive for COVID-19 nucleic acid were included in this study. We performed a retrospective review of records from January 2020 to April 2020. Inclusion criteria: (1) age>12, (2) all enrolled patients signed informed consent. Exclusion criteria: (1) pregnant or lactating women (2) severe cardiovascular diseases (3) drug allergy (4) infectious diseases. (5) abnormal liver and kidney functions. Patients were divided into two groups according to whether they had hypertension. Among them, 28 patients had hypertension and 102 patients did not. Patient laboratory tests and epidemiological characteristics were obtained from the hospital medical record system and used for analysis.

### Statistical method

The STATA version 12.0 software package (STATA Corp., College Station, TX, USA) was used to assess the collected data. The measurement data are presented as mean ± standard deviation (mean ± SD) and the enumeration data are expressed in numbers (percentages). The differences in continuous variables between the two groups were tested using one-way analysis of variance. Chi-square analysis or Fisher’s exact test was performed to compare the differences of categorical variables. A P value less than 0.05 was considered statistically significant.
